# Resolving Diagnostic Uncertainty in Neurodevelopmental Disorders Using Exome Sequencing Supported by Literature-Based Multi-Omics Evidence

**DOI:** 10.3390/biom16030399

**Published:** 2026-03-08

**Authors:** Danijela Krgovic, Peter Gradisnik, Andreja Osterc Koprivsek, Ana Kogovsek, Nadja Kokalj Vokac, Spela Stangler Herodez

**Affiliations:** 1 University Institute of Genetic Diagnostics, University Medical Centre Maribor, 2000 Maribor, Slovenia; 2 Department of Molecular Biology, Faculty of Medicine, University of Maribor, 2000 Maribor, Slovenia; 3 Department of Pediatrics, University Medical Centre Maribor, 2000 Maribor, Slovenia

**Keywords:** neurodevelopmental disorders, variant interpretation, whole-exome sequencing, multi-omics integration

## Abstract

*Background*: Neurodevelopmental disorders (NDDs) are genetically heterogeneous, and exome sequencing (ES) is now a first-line diagnostic tool. However, many patients receive variants of uncertain significance (VUSs) or inherited variants with incomplete penetrance, limiting clinical interpretation. Emerging multi-omics evidence from the literature can support the interpretation of novel and rare variants, helping to refine classification in selected cases. *Methods*: We assessed 20 patients and their parents referred for genetic testing for NDDs. ES was performed, followed by ACMG/ACGS-based variant classification, segregation analysis, and targeted literature review. Variants were included when deemed plausible contributors to the phenotype by a multidisciplinary team. Gene-level constraint metrics, in silico predictions, and emerging multi-omics evidence from the literature were integrated to support interpretation. *Results*: Across 18 NDD-associated genes, we identified 20 rare variants: Three pathogenic (P), nine likely pathogenic (LP), and eight VUSs. All P and most LP variants were de novo. Inherited variants, particularly in *KMT5B*, *TANC2*, *SPTBN1*, and *CHD4*, highlighted challenges related to incomplete penetrance. Two patients had dual molecular diagnoses. Several VUSs were supported by literature-derived transcriptomic, proteomic, or model-system evidence. *Conclusions*: This cohort underscores ongoing challenges in interpreting VUSs and inherited variants in NDDs. Integrating genomic findings with published multi-omics data enhances variant interpretation, reveals mechanistic insights, and strengthens diagnostic confidence, supporting broader adoption of multi-omics approaches in rare NDD evaluation.

## 1. Introduction

Neurodevelopmental disorders (NDDs) comprise a diverse group of conditions with substantial genetic contributions, yet for many individuals, the underlying etiology remains unclear. Considerable effort has been made to determine disease-related genes through trio-based exome or genome sequencing, where large studies identified de novo variants in probands [1,2,3]. These studies demonstrated that NDDs arise from disruptions in diverse biological pathways, yet many implicated genes converge on a limited number of core neurobiological processes.

Exome sequencing (ES) and genome sequencing (GS) are considered first-line diagnostic methods in NDDs with an estimated diagnostic yield by detecting pathogenic (P) or likely pathogenic (LP) variants in NDD-associated genes of 35–40% [4,5]. Despite significant contributions to the elucidation of the etiology of disease, exome or genome sequencing often leads to the identification of variants of uncertain significance (VUSs) and variants with possible incomplete penetrance, which represents a burden for both patients and healthcare professionals due to their unclear role in the disease [3,6]. According to recent clinical reviews, reanalysis of sequencing data—integrating updated clinical information, newly published evidence, and expanded variant databases—can lead to reclassification of variants and increase diagnostic yield by up to 12% [7,8]. However, VUSs remain a great challenge given the insufficient proof of their pathogenic effects, or they are “overlooked” during the analysis. Furthermore, incomplete penetrance in NDDs has already been associated with recurrent copy number variants (CNVs) that encompass multiple genes [6,9]. Yet, evidence for single-nucleotide variants (SNVs) is limited. A study of unaffected individuals from the UK Biobank demonstrated that rare, potentially deleterious variants in genes and loci associated with dominant monogenic developmental disorders can be found in adults from an unselected population cohort, where they are associated with a mild developmental phenotype [10]. Additionally, a recent study of twelve NDD patients showed they inherited a P or LP loss-of-function (LoF) variant from a phenotypically healthy parent. In six families where segregation was possible, the disease-causing variant was confirmed to be de novo in the healthy parent [6]. Identification of de novo variants through trio-based exome or genome sequencing has become a cornerstone of genetic evaluation in intellectual disability (ID) [1,2]. As a result, it is increasingly important to incorporate the concept of incomplete penetrance into variant interpretation frameworks and to approach inherited variants with caution [6]. Moreover, testing a broad set of genes allows us to identify potential variants in disease-causing genes where the genotype–phenotype does not fully match the clinical presentation of the patient, especially in genes rarely described in the literature.

There are several approaches for reducing the VUSs in diagnostics, from reanalysis of the obtained sequencing data to transcriptomics, proteomics and other multi-omics, which are increasingly used for improving diagnostic yield in NDDs and other rare disorders.

In this study, we describe a small cohort of NDD patients in whom plausibly disease-causing variants were identified through trio-based ES. We present both rare de novo and inherited VUSs, LP, and P variants and highlight how emerging multi-omics evidence from the literature can support the interpretation of novel and rare variants, helping us to refine their classification in selected cases.

## 2. Materials and Methods

### 2.1. Patients

Patients and, if available, their parents were recruited from University Medical Centre Maribor (Slovenia) in the years 2018 to 2025, who were referred to genetic diagnostics due to NDDs. The referring clinicians provided clinical information using the HPO (Human Phenotype Ontology) terms [11]. ES analysis was performed using a phenotype-driven analysis. Potentially disease-causing variants were classified according to the American College of Medical Genetics and Genomics (ACMG) and ACGS Best Practice Guidelines for Variant Classification in Rare Disease 2024 [12,13].

Written informed consent was signed by patients or their legal representative for the use of their medical history and genetic testing report.

Demographic and clinical characteristics of the patients enrolled in the study, including age, sex, and relevant medical history, are summarized in Supplementary Table S1.

### 2.2. Exome Sequencing

ES was performed on patients and in their parents, except for one patient, using DNA extracted from peripheral blood leukocytes with the QIAamp^®^ DNA Blood Midi Kit (QIAGEN, Hilden, Germany). Library constructions were performed with Illumina DNA Prep with Enrichment kit (Illumina, San Diego, CA, USA) and xGen™ Exome Hybridization Panel (IDT-Integrated DNA Technologies, Coralville, IA, USA) according to the manufacturer’s instructions. The DNA libraries were then sequenced on an Illumina HiSeq 6000 platform (150-bp paired-end reads) (Illumina, San Diego, CA, USA) with an average depth coverage of 100× with an average 12 Gb output per sample.

The obtained fastq data processing and annotation were performed using the commercially available bioinformatics tool Franklin (QIAGEN, Hilden, Germany), which was also used for SNV and CNV variant interpretation, establishing genotype–phenotype correlations, and a partial literature search.

### 2.3. Inclusion Criteria for Variants

Potentially disease-causing variants were subsequently subjected to additional manual classification following ACMG/ACGS criteria, accompanied by a targeted review of the relevant literature. Based on the aggregated clinical and molecular data, a multidisciplinary team of laboratory geneticists and treating physicians evaluated whether each variant should be considered reportable within the diagnostic setting. Only patients for whom the detected VUS or P/LP variant was determined as a plausible cause of the clinical phenotype were ultimately included in this study.

Inclusion criteria for SNVs comprised the following: classification as VUS, LP, or P according to ACMG/ACGS standards; an allele frequency below 0.00001 in the gnomAD v4.1.0 population database; computational evidence supporting a deleterious effect based on in silico prediction tools such as REVEL (REVEL v1.3) [14], AlphaMissense [15], or SpliceAI (SpliceAI v1.3.1) [16]; segregation analysis; de novo occurrence; or inheritance compatible with possible incomplete penetrance. Relevant published literature was additionally reviewed to support variant inclusion.

## 3. Results

Our study presents a cohort of 20 NDD patients referred to genetic testing in our facility, in whom ES trio analysis was performed, except for one patient, for whom the parents did not consent to the analysis. Patients whose VUS or P/LP variants did not account for the clear referral diagnosis were selected for this cohort.

ES trio analysis was performed in 19/20 patients. A total of 15 boys and five girls with an age range between 1 and 15 years were tested. The analysis identified three pathogenic (P) variants, nine likely pathogenic (LP), and eight VUSs. All P variants arose de novo in the proband. In three patients, the LP variants were inherited from an apparently healthy parent, in four they were de novo, and one patient inherited a variant from an affected mother. For one LP variant, the origin could not be determined. De novo VUSs were identified in three patients; the other five were inherited from the parents. We identified nine missense variants, 10 LoF variants, and one in-frame deletion.

In patients 13 and 20, additional variants were identified that were concordant with the reported clinical features, resulting in a dual molecular diagnosis.

In Table 1 and Table 2, clinical data and results from the ES analysis are presented. Patients are grouped by ACMG/ACGS classifications. Demographic and clinical characteristics of the enrolled patients, including age, sex, and relevant medical history, are provided in Supplementary Table S1.

There were 20 variants in 18 different genes, all known to be involved in the etiology of NDDs, that were identified. The affected genes converged on four main biological domains: the chromatin and transcriptional regulation (*ASH1L*, *KMT5B*, *CHD4*, *ZNF462*, *DDX3X*, *ANKRD17*), developmental signaling pathways (*CDON*, *PTCHD1*, *DLL1*, *CTNNB1*), synaptic structure and signaling (*SYNGAP1*, *TANC2*, *SPTBN1*, *VAMP2*, *CTNNB1*), and metabolism/autophagy (*TMLHE*, *WDFY3*). A summary of their biological roles at the cellular level is presented in Table 3.

**Table 1:** Variants of uncertain significance (VUSs) (Patients 1–8). This table summarizes eight VUSs identified in genes associated with NDDs. For each variant, detailed information is provided on inheritance, variant type, in silico prediction, affected protein domain, ACMG/ACGS criteria, phenotype–genotype match, and clinical interpretation. Most variants were missense changes located in functionally important domains, with partial phenotype overlap and insufficient evidence for pathogenicity. Detailed clinical characteristics of the enrolled patients are provided in Supplementary Table S1.

Patient	Gene	HGVS (cDNA/ Protein)	Zygosity	Inheritance	Variant Type	Key Clinical Features	gnomAD v4.1.0 Frequency	In Silico Prediction	Protein Domain	ACMG/ACGS Criteria	Phenotype–Genotype Match	Clinical Interpretation
1	*CDON*	c.737G>T p.(Ser246Ile)	het	dn	missense	Optic chiasm hypoplasia; cortical malformations; epilepsy; GDD	Absent	Uncertain	Extracellular Ig-like domain	PS2_sup, PM2_sup	Partial	Variant partial overlaps holoprosencephaly spectrum; uncertain contribution.
2	*PPP3CA*	c.1253_1258del p.(Glu418_Ser419del)	het	dn	in-frame deletion	ADHD; NDD; ID; tall stature	Absent	Likely damaging	Catalytic domain	PM2_sup, PM4_sup, PS2_sup	Partial	Variant affects key domain; phenotype milder than known DEE spectrum.
3	*KIF4A*	c.896A>G p.(Asp299Gly)	hemi	mat	missense	Mild GDD; autism; language delay	Absent	Possibly damaging	Motor domain	PM2_sup, PP3_mod	Partial	XL gene; phenotype overlaps; mat carrier unaffected.
4	*ASH1L*	c.5051G>A p.(Arg1684Gln)	het	pat	missense	NDD; autism	0.0000099	Damaging	SET domain	PM2_sup	Strong	Variant fits known ASD/NDD phenotype.
5	*KMT5B*	c.2138_2139del p.(Ser713TrpfsTer9)	het	mat	frameshift	GDD; autism; macrocephaly; speech delay	Absent	NMD not predicted	C-terminal region	PVS1_str, PM2_sup	Strong	Truncating variant matches known AD disorder.
6	*PTCHD1*	c.2317T>C p.(Ser773Pro)	hemi	mat	missense	ASD; hypotonia; speech delay	Absent	Possibly damaging	Transmembrane domain	PM2_sup, PP3_sup	Partial	Variant in ASD susceptibility gene; uncertain penetrance.
7	*TMLHE*	c.827A>G p.(Tyr276Cys)	hemi	mat	missense	ASD; GDD; speech delay	Absent for hemi	Possibly damaging	Enzyme active site	PM2_sup, PP3_sup	Partial	Variant may contribute to ASD susceptibility.
8	*ANKRD17*	c.6884A>T p.(Asp2295Val)	het	dn	missense	Autistic behavior; speech delay	Absent	Damaging	Ankyrin repeat	PM2_sup, PP2_sup, PS2_sup	Strong	Variant consistent with Chopra–Amiel–Gordon syndrome.

**Table 2:** Likely pathogenic (LP) (Patients 9–17) and pathogenic (P) variants (Patients 18–20). This table presents inherited and de novo P/LP variants identified in 12 individuals. The majority were truncating variants predicted to undergo nonsense-mediated decay (NMD), affecting critical protein domains. All variants showed strong phenotype–genotype concordance and aligned with known gene-specific neurodevelopmental syndromes. In silico predictions and ACMG/ACGS criteria consistently supported pathogenicity. Detailed clinical characteristics of the enrolled patients are provided in Supplementary Table S1.

Patient	Gene	HGVS (cDNA/Protein)	Zygosity	Inheritance	Variant Type	Key Clinical Features	gnomAD v4.1.0 Frequency	In-Silico Prediction	Protein Domain	ACMG/ACGS Criteria	Phenotype–Genotype Match	Clinical Interpretation
9	*TANC2*	c.4197dup p. (Lys1400Ter)	het	pat	frameshift insertion	GDD; delayed speech; delayed social milestones; ADHD; motor stereotypy; SGA	Absent	NMD predicted	TPR_8 C-terminal region	PVS1_vstr, PM2_sup	Strong	Truncating *TANC2* variants cause AD NDD with autistic features; phenotype highly consistent.
10	*SPTBN1*	c.951G>A p. (Trp317Ter)	het	pat	stop-gain	NDD; autism	Absent	NMD predicted	Spectrin repeat region	PVS1_vstr, PM2_sup	Strong	Classic *SPTBN1* phenotype; variant clearly pathogenic.
11	*DLL1*	c.845del p. (Gly282AlafsTer8)	het	mat	frameshift deletion	NDD; autism	Absent	NMD predicted	EGF-like domain	PVS1_vstr, PS4_sup, PM2_sup	Strong	*DLL1* truncating variants cause AD NDD; phenotype matches known disorder.
12	*CHD4*	c.3548G>A p.(Arg1183His)	het	mat	missense	NDD; facial dysmorphism; heart anomaly	Absent	Damaging	Helicase domain	PP1_mod, PP2_mod, PM5_mod, PP3_mod, PM2_sup	Strong	Variant fits Sifrim–Hitz–Weiss syndrome; phenotype highly consistent.
13	*VAMP2*	c.29dupC p.(Ala11CysfsTer19)	het	not tasted	frameshift insertion	DD; congenital stationary night blindness	0.000003	NMD predicted	N-terminal region	PVS1_vstr, PM2_sup	Strong	Truncating *VAMP2* variants cause AD NDD; phenotype consistent with known syndrome.
14	*ASH1L*	c.1621_1624del p.(Ser541ProfsTer9)	het	dn	frameshift deletion	Autistic behavior; dysmorphic facies	Absent	NMD predicted	N-terminal region	PVS1_vstr, PM2_sup, PS2_sup	Strong	Truncating *ASH1L* variant matches known AD NDD phenotype; highly consistent.
15	*KMT5B*	c.973G>A p.(Glu325Lys)	het	dn	missense	GDD; hypotonia	Absent	Damaging	Catalytic domain	PS4_mod, PS2_sup, PM2_sup, PP3_mod	Strong	Variant fits known *KMT5B*-related NDD; phenotype consistent.
16	*CTNNB1*	c.887_896del p.(Ile296ThrfsTer6)	het	dn	frameshift deletion	Microcephaly; axial hypotonia; GDD; facial dysmorphism	Absent	NMD predicted	Armadillo repeat	PVS1_vstr, PM2_sup, PS2_sup	Strong	*CTNNB1* truncating variants cause AD NDD with spastic diplegia; phenotype matches.
17	*DDX3X*	c.641T>A p. (Ile214Asn)	het	dn	missense	Craniosynostosis; plagiocephaly; facial anomalies; mild GDD	Absent	Damaging	Helicase ATP-binding domain	PM5_mod, PS2_sup, PP2_sup, PM2_sup, PP3_sup	Strong	Variant consistent with Snijders Blok syndrome; phenotype overlaps known spectrum.
18	*WDFY3*	c.256C>T p.(Arg86Ter)	het	dn	stop gain	Macrocephaly; hypotonia; motor delay; joint hypermobility	Absent	NMD predicted	N-terminal region	PVS1_vstr, PS2_sup, PM2_sup	Strong	Variant likely pathogenic; phenotype overlaps reported cases.
19	*SYNGAP1*	c.3706C>T p. (Gln1236Ter)	het	dn	stop gain	NDD; autism	Absent	NMD predicted	C-terminal region	PVS1_vstr, PM5_sup, PS2_sup, PM2_sup	Strong	Classic *SYNGAP1* phenotype; phenotype highly consistent.
20	*ZNF462*	c.429del p.(Val144SerfsTer5)	het	dn	frameshift deletion	Mild microcephaly; language delay; ASD; hearing impairment; GDD	Absent	N/A	N-terminal region	PVS1_vstr, PM2_sup, PS2_sup	Strong	Variant consistent with Weiss–Kruszka syndrome; phenotype highly consistent.

Abbreviations: GDD, global developmental delay; ADHD, attention-deficit/hyperactivity disorder; ASD, autism spectrum disorder; ID, intellectual disability; DD, developmental delay; NDD, neurodevelopmental delay; SGA, small for gestational age; het, heterozygous; hemi, hemizygous; dn, de novo; mat, maternal; pat, paternal; AD, autosomal dominant; XL, X-linked; VUS, variant of uncertain significance; LP, likely pathogenic; P, pathogenic; N/A, uncertain.

### 3.1. Patients with Present VUSs

#### 3.1.1. Patient 1

A 15-year-old girl with optic chiasm and cerebellar hypoplasia, cortical malformations, epilepsy, and GDD harbored a de novo missense variant in *CDON*. De novo missense changes in *CDON* are reported in the holoprosencephaly spectrum (HPE11; OMIM #614226) with variable severity [17,18]. The patient’s malformations (optic pathway anomalies and cortical malformations) may co-occur with midline brain malformations but are not considered part of HPE per se and reflect distinct developmental mechanisms with partial pathway overlap [19].

In silico tools (REVEL [14], AlphaMissense [15], SpliceAI [16]) did not predict a pathogenic effect; the variant was absent from gnomAD v4.1.0 and occurred de novo.

#### 3.1.2. Patient 2

A 7-year-old boy with attention-deficit/hyperactivity disorder (ADHD), intellectual disability (ID), increased body weight, and tall stature carried a de novo 6 bp in-frame deletion in the *PPP3CA* regulatory domain (RD). Pathogenic *PPP3CA* variants produce two phenotypes: GoF missense variants in the autoinhibitory domain (AID) linked to arthrogryposis, cleft palate, craniosynostosis, and impaired intellectual development [20]; and LoF variants in the catalytic domain (CD) associated with developmental and epileptic encephalopathy 91 [21,22]. The functional impact of RD variants remains uncertain; truncating RD variants have been associated with more severe epilepsy [23], which was not present here.

#### 3.1.3. Patient 3

A 6-year-old boy with mild GDD, language impairment, and autistic features had a maternally inherited hemizygous missense variant in the *KIF4A* kinesin motor domain. Published *KIF4A* NDD reports remain limited; the phenotype aligns with previously described individuals with DD and limited speech without epilepsy [24]. The variant is unreported and was classified as a VUS (ACMG/ACGS).

#### 3.1.4. Patient 4

A 6-year-old girl with DD and autistic features carried an inherited *ASH1L* missense variant. De novo LoF variants in *ASH1L* are implicated in autosomal dominant NDD with a broad spectrum [25,26]. While missense variants in *ASH1L* have been reported, they have thus far been exclusively de novo, and their pathogenic relevance has not been fully established [26,27].

Patient 4 has a notable family history of delayed speech-language development in her older brother and behavioral difficulties reported in a paternal cousin.

This paternally inherited missense change is rare (gnomAD v4.1.0: 0.001%) and was classified as VUS (ACMG/ACGS PM2_sup). MetaDome and gene constraint (Z = 3.47) provide supportive but insufficient evidence for pathogenicity.

#### 3.1.5. Patient 5

A 4-year-old boy with GDD, autistic features, delayed speech, motor stereotypies, tiptoe gait, macrocephaly, and drooling had a maternally inherited *KMT5B* frameshift deletion. The phenotype aligns with the clinical spectrum reported by Sheppard et al. using human cohorts, knockout mice, and functional assays [28]. Most reported pathogenic variants are de novo or inherited from affected parents, though rare LoF carriers without overt phenotype have been noted [28]. This observation prompts consideration of whether mild or previously unrecognized parental features may be present, or whether incomplete penetrance may occur for pathogenic *KMT5B* variants. Namely, variable expressivity/incomplete penetrance across NDD genes has been increasingly recognized [6].

#### 3.1.6. Patient 6

A 12-year-old boy with ASD had a maternally inherited missense variant in *PTCHD1*. Truncating mutations/microdeletions have been associated with X-linked autism-4 (AUTS4) [29] and supported by animal studies [30]. Functional studies indicate that some missense variants, especially in extracellular loops or transmembrane regions, impair protein stability/membrane localization [31,32,33]. Per current criteria, the variant was classified as VUS.

#### 3.1.7. Patient 7

A 3-year-old boy with ASD, DD, and delayed speech/language had a maternally inherited missense variant in *TMLHE*. Missense variants are less frequently reported and lack functional characterization [34]. The variant was seen in one female in gnomAD v2.1.1 and not in the newer release; to our knowledge, it has not been reported in ASD. In silico predictions suggest potential damage.

#### 3.1.8. Patient 8

A de novo missense variant in *ANKRD17* was identified in a 7-year-old boy referred for evaluation of suspected autistic behavior and noticeable speech delay. *ANKRD17* has only recently been implicated in ID and ASD [35]. Data from a large patient cohort, supported by single-cell RNA-seq analyses, suggests that haploinsufficiency may represent the underlying disease mechanism. By using protein modelling, authors predicted that the majority of missense variants affect the stability of ankyrin repeat domains [36].

In this context, the variant identified in our patient is likely to have a similar functional impact and may therefore explain his clinical presentation.

### 3.2. Patients with Inherited LP Variants

#### 3.2.1. Patient 9

A 3-year-old girl with GDD, marked speech/language impairment, delayed motor/social milestones, motor stereotypies, and ADHD traits had a paternally inherited *TANC2* frameshift deletion. *TANC2* shows expression in the developing human brain, and animal knockout models show NDD-relevant phenotypes [37,38,39]. Although many reported variants are de novo, autosomal dominant inheritance is documented [37].

#### 3.2.2. Patient 10

A 2-year-old boy with DD and ASD carried a paternally inherited premature stop-gain in *SPTBN1*. *SPTBN1* variation is associated with an autosomal dominant NDD with a broad phenotypic spectrum; most variants are de novo, with few inherited from affected parents [40,41,42]. LoF variants have also been implicated in an early-onset distal myopathy with chronic neurogenic features [43].

#### 3.2.3. Patient 11

A 4-year-old boy with DD and ASD had a heterozygous LP frameshift deletion in *DLL1*, inherited from an unaffected mother. A duplication at the same nucleotide (DLL1:c.845dup) is present in the ClinVar database (Variation ID 1098334). *DLL1* was implicated in NDDs with a mix of de novo and dominantly inherited variants [44].

#### 3.2.4. Patient 12

This patient represents the only case in our cohort with an inherited variant, with the mother exhibiting a clinical phenotype similar to her child. An LP missense variant in *CHD4* was identified in a 9-year-old boy and his mother. Clinical evaluation of the child revealed neurodevelopmental delay, craniofacial dysmorphism, and abnormal cardiac morphology. Family history is notable for a brother with a perinatal leg injury, a sister with high myopia and strabismus, a mother with a history of childhood cardiac anomaly, special-education schooling, and psychiatric treatment, and a father with type 2 diabetes.

Sifrim–Hitz–Weiss syndrome (SIHIES) is a multisystem NDD caused by de novo pathogenic variants in *CHD4,* where patients show GDD, mild to moderate ID, brain anomalies, congenital heart defects and dysmorphic features [45].

#### 3.2.5. Patient 13

An LP heterozygous frame-shift insertion in the *VAMP2* gene was identified in a 12-year-old boy with DD and congenital stationary night blindness. This patient has an additional variant: a homozygous stop gain mutation in the *TRPM1* gene (*TRPM1*:c.2684G>A; p.(Trp.895Ter)). Segregation analysis was not performed for this patient.

*VAMP2* was established to be a genetic cause of NDD with hypotonia and autistic features with or without hyperkinetic movements in a small cohort of five unrelated patients [46]. To this date, only a handful of additional patients with LoF, in-frame deletions, and missense variants in the *VAMP2* gene have been reported [47]. Our Patient 13 is thus one of the rare cases with frame-shift insertion in this gene.

Patient 13 is one of two patients in our cohort with a dual diagnosis. Specifically, a homozygous pathogenic variant in *TRPM1* accounts for the presence of autosomal recessive congenital stationary night blindness (CSNB) (OMIM #613216).

### 3.3. Patients with De Novo P/LP Variants

#### 3.3.1. Patient 14

An 8-year-old boy with autistic features and dysmorphic facial features carried a de novo frameshift deletion in *ASH1L*, consistent with autosomal dominant NDD associated with ASH1L LoF [25,26].

#### 3.3.2. Patient 15

A de novo, likely pathogenic missense variant in *KMT5B* was identified in a 7-year-old boy presenting with GDD and hypotonia. His clinical presentation appears milder than that of Patient 5; however, published data indicate that both missense and putative LoF *KMT5B* variants are associated with reduced cellular growth in patient-derived cell models [28].

#### 3.3.3. Patient 16

A de novo frame-shift deletion of 7 bp was determined in *CTNNB1* in a 1-year-old patient with craniofacial anomalies, microcephaly, hyperreflexia, DD, and axial hypotonia.

A systematic review of published cases of *CTNNB1* syndrome has previously been conducted to assess potential genotype–phenotype correlations. This analysis confirmed substantial genotypic and phenotypic heterogeneity and suggested that LoF variants, including those located in exon 6—as observed in our patient—are associated with more severe clinical presentations [48].

#### 3.3.4. Patient 17

In a 1-year-old girl with plagiocephaly, craniosynostosis, facial dysmorphism, and mild DD, an LP de novo missense variant was tested in the *DDX3X* gene.

This gene is primarily associated with an X-linked dominant ID disorder, although rare X-linked recessive inheritance has also been reported in males. By using in vitro assays and zebrafish models, the authors also showed that several missense variants disrupt canonical WNT/β catenin signaling, consistent with the LoF mechanism. No evidence of a dominant negative effect was found, and the data support haploinsufficiency as the likely pathogenic mechanism [49]. More recent evidence indicates that de novo *DDX3X* variants are not necessarily male lethal and can be a cause of syndromic ID in both males and females [50].

The variant identified in our Patient 17 was not included in this study, underscoring the need for additional functional investigations.

#### 3.3.5. Patient 18

A 2-year-old boy with macrocephaly, delayed gross motor development, joint hypermobility, and hypotonia had a de novo premature stop in *WDFY3* (exon 5/68). *WDFY3* LoF has been associated with mild–moderate NDD, psychiatric features, and macrocephaly; variants in the PH domain with possible GoF have been linked to microcephaly [51,52,53].

The family history is notable for maternal connective-tissue laxity similar to that observed in her child; however, no associated genetic variants were identified.

#### 3.3.6. Patient 19

A de novo premature stop codon variant in the *SYNGAP1* gene was identified in a 2-year-old boy presenting with NDD and autism. *SYNGAP1*-related disorder, historically referred to as intellectual developmental disorder-5 (MRD5), is an autosomal dominant condition characterized by moderate to severe IDD with delayed psychomotor development. Additional clinical features are frequently observed, and ASD is commonly reported in affected individuals [54,55].

#### 3.3.7. Patient 20

Patient 20 represents the second individual in our cohort with a dual molecular diagnosis. In this 4-year-old girl presenting with mild microcephaly, language delay, ASD, and hearing impairment, we identified a de novo P frameshift deletion in *ZNF462*. In addition, two heterozygous P *GJB2* variants were detected: *GJB2*:c.269T>C (maternally inherited) and *GJB2*:c.35del (paternally inherited).

Individuals with *ZNF462* LoF variants are diagnosed with autosomal dominant Weiss–Kruszka syndrome [56]. Although the condition is increasingly recognized as additional cases are reported, its clinical spectrum is still being defined. The syndrome is characterized by DD, ASD, and distinctive craniofacial features, with microcephaly, speech delay, and hearing impairment also frequently observed [56,57,58,59].

**Table 3:** Biological functions of investigated genes and multi-omics evidence. This table compiles functional data for all genes represented in the cohort, including evidence from animal models, transcriptomics, proteomics, chromatin studies, and cellular assays published in the literature. These data provide essential biological context for variant interpretation.

Genes	Biological Functions of Investigated Genes in Etiology of NDD
***ANKRD17***	*ANKRD17* encodes ankyrin repeat domain containing protein 17, which encourages cell cycle progression by interacting with cyclin E/CDK2. In vitro studies suggest it also contributes to innate immune responses. *Ankrd17* knockout mice display abnormal blood vessel formation and hemorrhage, leading to lethality by embryonic day 11, limiting the ability to study the gene’s function. Its underlying molecular mechanisms in NDD are still unknown [35,51].
***ASH1L***	*ASH1L* encodes a histone methyltransferase that catalyzes H3K4 and H3K36 methylation and has an important role in chromatin modification and gene transcription. []. Loss of ASH1L in mouse brain models alters expression of synaptic and neuronal of *ASH1L*-mediated genes and leads to neurodevelopmental phenotypes associated with ASD and ID, suggesting influence on neural gene regulation and synaptic gene expression [60,61,62].
***CDON***	*CDON* encodes an Ig superfamily receptor that plays an important role in cell–cell adhesion and developmental signal transduction, and it regulates the differentiation as co-receptor in the Hedgehog pathway [63]. Shh^−/−^ mice develop midline, brain, limb, and skeletal anomalies, including cyclopia and missing vertebrae and ribs [64,65].
***CHD4***	*CHD4* encodes a chromodomain helicase DNA-binding protein 4, which is a remodeling protein involved in epigenetic regulation of gene transcription, DNA repair, and cell cycle progression [66]. Knockout of *Chd4* in granule neurons of the mouse cerebellum showed that Chd4 loss in the brain disrupts normal genome architecture, pushing normally repressed regions into a more active state. Dysregulated genome architecture represents a main mechanism of how chromatin regulators lead to NDD [66,67].
***CTNNB1***	*CTNNB1* encodes βcatenin, which is a core component of the chadherin complex and plays an important role in stem cell renewal, cell proliferation and differentiation during embryogenesis [68,69]. While GoF mice models show overactivation of βcatenin signaling connected to oncogenesis and some behavioral phenotypes, LoF mice models reveal the essential role of βcatenin in proper neurodevelopment [70].
***DDX3X***	*DDX3X* encodes RNA helicase, which has a key role in mRNA translation [71]. Dysregulation of mRNA during brain development has been reported in mouse models of NDD [72], which was also observed in Ddx3x haploinsufficient female mice [73].
***DLL1***	*DLL1* encodes a Notch ligand that binds to Notch receptors and activates intracellular signaling, which is essential for developmental processes [74]. The study in knockout mice showed that *Dll1* haploinsufficiency increases the risk of brain abnormalities with functional impact [75].
***KIF4A***	*KIF4A* encodes a kinesin-4 family motor protein that regulates cell cycle processes, including PRC1-dependent central spindle organization and cytokinesis, modulates neuronal survival, and is necessary for balanced synaptic transmission and normal neuronal development. [24]. A *Kif4a* knock-in mouse showed abnormal neuronal morphology, DD, and a lower seizure threshold [76].
***KMT5B***	*KMT5B* encodes a histone H4K20 methyltransferase. *Kmt5b* haploinsufficient mouse brains showed altered expression of pathways involved in nervous system development and function, including those regulating axon guidance signaling [28,77], and its deficiency is linked to NDD [78].
***PPP3CA***	*PPP3CA* encodes a calcium-activated phosphatase that connects Ca^2+^ signals to phosphorylation changes affecting transcription and synaptic function [21]. LoF and GoF variants in *PPP3CA* cause two separate clinical syndromes [20].
***PTCHD1***	*PTCHD1* encodes a transmembrane protein with a patched-like domain, which is important for proper synaptic function and neurodevelopmental. Loss of *Ptchd1* in male mice results in excitatory synaptic dysfunction [79]. *Ptchd1* knockout mice show abnormal behavior caused by weaker excitatory signals and changed dendrites. Similar findings were obtained in human-derived neuronal models [33].
***SPTBN1***	*SPTBN1* encodes βII-spectrin, which forms micrometer-scale networks associated with plasma membranes [40]. The knockout mice for neuronal βII-spectrin have defects in cortical organization, DD, and behavioral deficiencies [80].
***SYNGAP1***	*SYNGAP1* encodes a synaptic Ras GTPase-activating protein that plays a key role in synaptic plasticity [81]. *SYNGAP1* haploinsufficiency in human cortical organoids and a complementary mouse model revealed disrupted cytoskeletal control in radial glia that alters progenitor–neuron balance and accelerates cortical neuron maturation, pointing to a non-synaptic mechanism in *SYNGAP1*-related NDD [81,82].
***TANC2***	*TANC2* encodes a multi-domain postsynaptic scaffold protein that interacts with multiple postsynaptic density proteins [37]. The *Tanc2* knockout allele in zebrafish increases the larval brain size and body length due to increased proliferation and inhibited apoptosis. In adulthood, zebrafish showed signs equivalent to those of autism spectrum in humans [39]. Similar findings were observed in the mouse model [83].
***TMLHE***	*TMLHE* encodes an enzyme in carnitine biosynthesis that is required for fatty acid transport into mitochondria, and its deficiency was defined as a risk factor for autism [84,85]. On the contrary, in *Tmlhe* knockout mice, no significant social, cognitive, or repetitive-behavior changes were observed [86].
***VAMP2***	*VAMP2* encodes a v-SNARE protein involved in synaptic vesicle fusion and neurotransmitter release at the presynaptic level [46,87]. Knockout *Vamp2*^−/−^ mice die after birth due to severely decreased synaptic vesicle fusion, while *VAMP2*-deficient mice showed abnormalities in synaptic-vesicle morphology and size [46].
***WDFY3***	*WDFY3* encodes the ALFY protein, which regulates selective autophagy of protein aggregates and synaptic components. Loss of *Wdfy3* in mice causes selective cortical enlargement, mirroring the early brain overgrowth often observed in children with ASD [88].
***ZNF462***	*ZNF462* encodes zinc finger protein 462, which is implicated in gene expression regulation and chromatin remodeling. Mouse studies using a *Zfp462* knockout mouse (*ZNF462* murine homologue) model showed that Zfp462 silences meso-endodermal genes and acts as a safeguard for neural lineage specification of mouse embryonic stem cells. While knockout mice showed prenatal lethality, the heterozygous mice exhibited DD and anxiety-like behavior [56,57].

Abbreviations: NDD, neurodevelopmental delay; ASD, autism spectrum disorder; ID, intellectual disability; DD, developmental delay; LoF, loss of function; GoF, gain of function.

## 4. Discussion

NDDs arise from diverse genetic mechanisms and display substantial phenotypic heterogeneity, making variant interpretation a persistent challenge in clinical genomics. ES, particularly in trio settings, has become a cornerstone for identifying pathogenic de novo variants and distinguishing them from inherited background variation [1,2,3]. However, the classification of rare missense variants and VUSs remains difficult, especially in genes with reduced penetrance, variable expressivity, or limited functional evidence. Traditional ACMG/AMP criteria emphasize variant-centric evidence, often underweighting gene and pathway-level biological context. As a result, biologically plausible variants may remain formally “uncertain” despite compelling mechanistic clues.

This challenge is compounded by the limitations of animal models, which frequently fail to recapitulate the full spectrum of human neurodevelopmental phenotypes [89,90,91]. Multi-omics approaches—including transcriptomics, proteomics, epigenomics, and single-cell analyses—are increasingly essential for resolving genotype–phenotype discrepancies and refining disease mechanisms. The present cohort illustrates these complexities across three major groups: patients with VUSs (Table 1), patients with inherited and de novo P/LP variants (Table 2), and gene-level functional evidence from published multi-omics and animal studies (Table 3) provides critical context for interpreting these findings.

### 4.1. Variants of Uncertain Significance (Patients 1–8)

#### 4.1.1. Patient 1—CDON

De novo missense variant p.(Ser246Ile) affects the extracellular Ig-like domain and shows uncertain in silico prediction. Although *CDON* is a known holoprosencephaly gene, the patient’s phenotype—optic chiasm hypoplasia, cortical malformations, epilepsy, and GDD—only partially overlaps the HPE spectrum. *CDON* regulates Hedgehog (Hh) signaling in early eye development [92], consistent with the optic anomalies, but the absence of HPE features limits interpretability. *CDON*’s role in cell–cell adhesion and Hh signaling (Table 3) supports biological plausibility, yet the clinical contribution remains uncertain.

#### 4.1.2. Patient 2—PPP3CA

De novo in-frame deletion p.(Glu418_Ser419del) affects the catalytic domain and is predicted to be damaging. *PPP3CA* encodes a calcium-activated phosphatase linking Ca^2+^ signals to transcription and synaptic function [21]. Distinct phenotypes arise from GoF and LoF variants in different domains [20,21,22,23]. The regulatory domain (RD) remains poorly understood, and the absence of epilepsy in this patient complicates interpretation [23]. The variant’s partial phenotype match and uncertain functional consequences justify its VUS classification.

#### 4.1.3. Patient 3—KIF4A

The hemizygous missense variant p.(Asp299Gly) affects the motor domain and is predicted to be possibly damaging. *KIF4A* regulates cytokinesis, neuronal survival, and synaptic transmission [24]. Mouse models show abnormal neuronal morphology and reduced seizure threshold [76]. Despite partial phenotype overlap [24], the lack of prior reports and limited functional evidence support a VUS classification.

#### 4.1.4. Patient 4—ASH1L

The paternally inherited missense variant p.(Arg1684Gln) affects the SET domain and is predicted to be damaging. *ASH1L* encodes a histone methyltransferase that regulates H3K4/H3K36 methylation and neuronal gene expression [60,61,62]. Although the patient’s phenotype aligns with known *ASH1L*-related NDDs, all pathogenic missense variants reported to date have been de novo [26,27]. The low population frequency and elevated Z score provide supportive but insufficient evidence for pathogenicity. Notably, Patient 4 has a family history of delayed speech language development in an older brother and behavioral difficulties in a paternal cousin, features that may reflect variable expressivity but remain too nonspecific to clarify the variant’s contribution.

#### 4.1.5. Patient 5—KMT5B

The maternally inherited frameshift p.(Ser713TrpfsTer9) affects the C-terminal region and is predicted to escape NMD. *KMT5B* encodes a histone H4K20 methyltransferase essential for neurodevelopment [28,77,78]. Although truncating variants typically cause autosomal dominant NDD, rare unaffected carriers have been reported [28]. This case underscores the challenge of incomplete penetrance and highlights the importance of systematic parental phenotyping.

#### 4.1.6. Patient 6—PTCHD1

Functional studies increasingly show that missense variants in extracellular loops or transmembrane regions can destabilize *PTCHD1* and impair membrane localization [31,32,33]. The hemizygous p.(Ser773Pro) variant in our patient falls within such a transmembrane region, is predicted to be damaging, and lies in a domain where missense changes frequently compromise protein stability [31,32,33]. Given *PTCHD1*’s role in synaptic regulation—supported by knockout models demonstrating excitatory synaptic dysfunction and altered dendritic morphology [33,79]—disruption of protein stability may be relevant, although current evidence remains insufficient for definitive classification. Accordingly, the variant is considered as VUS, with partial phenotype overlap but uncertain penetrance supporting this designation. Emerging functional approaches, including CRISPR-based editing and iPSC-derived neuronal models, are becoming essential for resolving the impact of such variants, as illustrated by the characterization of another *PTCHD1* VUS (*PTCHD1*:c.2489T>G) in a child with ASD [93].

#### 4.1.7. Patient 7—TMLHE

The missense variant p.(Tyr276Cys) affects the enzyme’s active site and is predicted to be possibly damaging. Although *TMLHE* deficiency has been proposed as a risk factor for ASD [84,85], knockout mouse models do not exhibit ASD-like phenotypes [86], suggesting that such variants may contribute to susceptibility but remain insufficient for pathogenic classification. A similar pattern emerges for the *TMLHE* exon 2 deletion, which has been proposed as an autism susceptibility factor yet has also been identified in unaffected males [94]. In our cohort, this deletion was observed in two additional individuals: an 11-year-old girl with learning difficulties and her 8-year-old brother with autism, both of whom inherited the variant from an unaffected mother. Consistent with the uncertainty surrounding its pathogenic relevance, recent mouse studies indicate that knockout of this exon does not produce an ASD-like phenotype [86]. Together, these findings highlight the need for further functional and model-based studies to clarify the contribution of this and other *TMLHE* variants to neurodevelopmental phenotypes.

#### 4.1.8. Patient 8—ANKRD17

The de novo missense variant p.(Asp2295Val) affects an ankyrin repeat and is predicted to be damaging. *ANKRD17* knockout mice exhibit embryonic lethality and vascular defects [35,51], and data from a large patient cohort, supported by single-cell RNA-seq analyses, suggests that haploinsufficiency may represent the underlying disease mechanism. By using protein modeling, authors predicted that the majority of missense variants affect the stability of ankyrin repeat domains [36]. The strong phenotype match supports likely pathogenicity, but limited case numbers justify cautious interpretation.

### 4.2. Inherited Likely Pathogenic Variants (Patients 9–13)

#### 4.2.1. Patient 9—TANC2

The paternal frameshift p.(Lys1400Ter) affects the C-terminal TPR_8 region and is predicted to undergo NMD. *TANC2* encodes a postsynaptic scaffold protein essential for synaptic architecture [37], and knockout models demonstrate increased brain size, altered apoptosis, and ASD-like behaviors [39,83], providing strong phenotypic support for pathogenicity. Although many reported variants in *TANC2* are de novo, autosomal dominant inheritance has also been documented [37], consistent with the inheritance pattern observed here. Taken together, these findings reinforce the functional relevance of *TANC2* disruption and support the classification of this variant as likely pathogenic.

#### 4.2.2. Patient 10—SPTBN1

The paternal stop gain p.(Trp317Ter) affects the spectrin repeat region and is predicted to undergo NMD. *SPTBN1* is widely expressed across tissues [95], and knockout models show cortical disorganization and behavioral abnormalities [80]. The variant fits the classic *SPTBN1* phenotype.

#### 4.2.3. Patient 11—DLL1

The maternal frameshift p.(Gly282AlafsTer8) affects the EGF-like domain and is predicted to undergo NMD. *DLL1* encodes a Notch ligand essential for developmental signaling [74]. Haploinsufficiency in mice leads to brain abnormalities [75]. The phenotype strongly matches known *DLL1*-related NDDs.

#### 4.2.4. Patient 12—CHD4

The maternal missense variant p.(Arg1183His) affects the helicase domain and is predicted to be damaging. *CHD4* plays a key role in chromatin remodeling and genome architecture [66,67], and recent work has identified a DNA methylation episignature specific to pathogenic missense variants within this domain [96], providing additional support for pathogenicity. The family history includes a brother with a perinatal leg injury, a sister with high myopia and strabismus, and a mother with a childhood cardiac anomaly, special education schooling, and psychiatric treatment. Although these features are heterogeneous and not specific to *CHD4*-related disorders, they highlight a background of variable neurodevelopmental and medical traits within the family that may intersect with the observed variant’s effects, underscoring the need for further segregation analysis.

#### 4.2.5. Patient 13—VAMP2

The frameshift variant p.(Ala11CysfsTer19) affects the N-terminal region of *VAMP2* and is predicted to trigger NMD, consistent with a LoFtion mechanism. *VAMP2* plays a critical role in synaptic vesicle fusion and neurotransmitter release [46,87], and complete LoF in mouse models results in perinatal lethality due to severely impaired synaptic transmission [46]. The patient’s neurodevelopmental phenotype aligns well with previously described *VAMP2*-related NDDs. A second diagnosis (*TRPM1-*related CSNB) explains the night blindness.

Segregation analysis was not available for this case, which limits definitive interpretation. However, data from the DECIPHER database indicate that *VAMP2* is highly intolerant to LoF variation, with a pLI score of 1 (pLI ≥ 0.9), further supporting the pathogenic relevance of this variant.

### 4.3. De Novo Pathogenic/Likely Pathogenic Variants (Patients 14–20)

#### 4.3.1. Patient 14—ASH1L

The de novo frameshift p.(Ser541ProfsTer9) affects the N-terminal region and is predicted to undergo NMD. *ASH1L* LoF variants cause autosomal dominant NDD with ASD and dysmorphism [25,26]. The phenotype is highly consistent.

#### 4.3.2. Patient 15—KMT5B

The de novo missense p.(Glu325Lys) affects the catalytic domain and is predicted to be damaging. Both missense and LoF variants impair cellular growth in patient-derived models [28]. The phenotype matches known *KMT5B-*related NDD.

#### 4.3.3. Patient 16—CTNNB1

The de novo frameshift p.(Ile296ThrfsTer6) affects the armadillo repeat and is predicted to undergo NMD. *CTNNB1* encodes β catenin, a protein essential for neurodevelopment [70], and multi-omics studies have highlighted downstream pathway dysregulation [97]. A systematic review of *CTNNB1* syndrome cases has shown substantial genotypic and phenotypic heterogeneity and suggested that loss-of-function variants—including those in exon 6, as in our patient—are associated with more severe clinical presentations [48]. *CTNNB1*-deficient animal models further underscore the gene’s role in disease mechanisms and the need for detailed region and cell-specific analyses of neural circuitry [70]. High-resolution approaches such as proteomics, RNA seq, and ATAC seq have been proposed to identify downstream molecular changes and therapeutic targets [97]. Notably, therapeutic development has advanced to the creation of URBAGEN, the first gene replacement therapy specifically designed for *CTNNB1* syndrome [98].

#### 4.3.4. Patient 17—DDX3X

The de novo missense p.(Ile214Asn) affects the helicase ATP-binding domain and is predicted to be damaging. *DDX3X* regulates mRNA translation [71], and multi-omics studies show variant-specific effects on neurogenesis and RNA metabolism [99]. The phenotype overlaps with Snijders Blok syndrome.

#### 4.3.5. Patient 18—WDFY3

The de novo stop gain p.(Arg86Ter) affects the N-terminal region and is predicted to undergo NMD. *WDFY3* regulates selective autophagy of synaptic components [88]. Haploinsufficiency causes cortical enlargement and ASD-like features [51,53].

#### 4.3.6. Patient 19—SYNGAP1

The de novo stop gain variant p.(Gln1236Ter) affects the C-terminal region and is predicted to undergo NMD. *SYNGAP1* regulates synaptic plasticity [81], and multi-omics studies in organoids and mouse models demonstrate disrupted cytoskeletal control and accelerated neuronal maturation [81,82]. Although many individuals with *SYNGAP1-*related disorders have been described, the mechanisms by which *SYNGAP1* LoF alters human neuronal development and synaptic physiology remain under active investigation [100]. To address this, multiple experimental systems have been employed, including animal models that recapitulate conserved *SYNGAP1*-related phenotypes [101], iPSC-derived human neurons that model synaptic dysfunction in vitro [81], and human cortical organoids that enable analysis of variant-specific effects on neuronal maturation and circuit development [82,102,103]. Together, these complementary approaches aim to clarify how distinct *SYNGAP1* variants disrupt synapse structure and function.

#### 4.3.7. Patient 20—ZNF462

The de novo frameshift p.(Val144SerfsTer5) affects the N-terminal region of *ZNF462*, a gene that regulates chromatin remodeling and neural lineage specification [56,57]. The patient’s presentation is consistent with Weiss–Kruszka syndrome, an autosomal dominant condition caused by *ZNF462* LoF variants [56]. Although the number of reported cases is increasing, the full clinical spectrum is still being defined; characteristic features include developmental delay, autism spectrum disorder, and distinctive craniofacial findings, with microcephaly, speech delay, and hearing impairment also frequently described [56,57,58,59]. The molecular function of *ZNF462* remains incompletely understood, but recent work shows that its murine homolog preserves neural lineage identity by recruiting the G9A/GLP H3K9 methyltransferase complex to repress non-neural gene programs. Loss of *ZFP462* leads to aberrant activation of alternative lineage pathways, providing a plausible mechanistic basis for *ZNF462*-associated neurodevelopmental pathology [104].

## 5. Conclusions

This study demonstrates both the diagnostic strengths and the persistent limitations of WES in NDDs. While P and LP variants could be interpreted with confidence through convergence of clinical presentation, protein domain context, in silico predictions, and published multi-omics evidence, the VUS cases highlight ongoing challenges related to incomplete functional data, variable penetrance, and variant-specific effects. Importantly, the cohort also underscores difficulties specific to inherited LP variants, where subtle or unrecognized parental phenotypes and incomplete penetrance complicate interpretation and can obscure true genotype–phenotype relationships.

Although we did not generate multi-omics data ourselves, a systematic review of published transcriptomic, proteomic, epigenomic, and single-cell studies proved essential for contextualizing variant effects and refining mechanistic understanding across numerous genes in this cohort. Insights from Table 1, Table 2 and Table 3 highlight the importance of domain-aware variant interpretation, gene-level functional evidence, and careful phenotyping of carrier parents.

Continued progress will depend on expanding functional assays, developing patient-derived cellular and organoid models, and integrating multi-omics frameworks to refine genotype–phenotype correlations. As these approaches mature, they will improve diagnostic precision, support variant reclassification, and advance our broader understanding of NDD biology.

## Data Availability

The original contributions presented in this study are included in the article/supplementary material. Further inquiries can be directed to the corresponding author.

## Supplementary Materials

The following supporting information can be downloaded at: https://www.mdpi.com/article/10.3390/biom16030399/s1. Table S1: Demographic and clinical characteristics of the patients enrolled in the study.

## Abbreviations

The following abbreviations are used in this manuscript:

ADHD	Attention-deficit/hyperactivity disorder
ASD	Autism spectrum disorders
CNV	Copy number variant
CSNB	Congenital stationary night blindness
DD	Developmental delay
ES	Exome sequencing
GDD	Global developmental delay
GS	Genome sequencing
GoF	Gain-of-Function
HPO	Human Phenotype Ontology
ID	Intellectual disability
LoF	Loss-of-Function
LP	Likely pathogenic
MRD5	Intellectual developmental disorder-5
NDD	Neurodevelopmental disorders
OMIM	Online Mendelian Inheritance in Man
P	Pathogenic
SNV	Single-nucleotide variants
VUS	Variant of uncertain significance
